# Mirizzi Syndrome: Diagnosis and Management of a Challenging Biliary Disease

**DOI:** 10.1155/2018/6962090

**Published:** 2018-08-12

**Authors:** Gennaro Clemente, Andrea Tringali, Agostino M. De Rose, Elena Panettieri, Marino Murazio, Gennaro Nuzzo, Felice Giuliante

**Affiliations:** ^1^Department of Surgical Sciences, Hepatobiliary Unit, Fondazione Policlinico Universitario A. Gemelli IRCCS, Catholic University of Sacred Hearth, Rome, Italy; ^2^Department of Surgical Sciences, Surgical Endoscopy Unit, Fondazione Policlinico Universitario A. Gemelli IRCCS, Catholic University of Sacred Hearth, Rome, Italy

## Abstract

**Background:**

Mirizzi syndrome is a condition difficult to diagnose and treat, representing a particular “challenge” for the biliary surgeon. The disease can mimic cancer of the gallbladder, causing considerable diagnostic difficulties. Furthermore, it increases the risk of intraoperative biliary injury during cholecystectomy. The aim of this study is to point out some particular aspects of diagnosis and treatment of this condition.

**Methods:**

The clinical records of patients with Mirizzi syndrome, treated in the last five years, were reviewed. Clinical data, cholangiograms, preoperative diagnosis, operative procedures, and early and late results were examined.

**Results:**

Eighteen consecutive patients were treated in the last five years. Presenting symptoms were jaundice, pain, and cholangitis. Preoperative diagnosis of Mirizzi syndrome was achieved in 11 patients, while 6 had a diagnosis of gallbladder cancer and 1 of Klatskin tumor. Seventeen patients underwent surgery, including cholecystectomy in 8 cases, bile duct repair over T-tube in 3 cases, and hepaticojejunostomy in 4 cases. Two cases (11.1%) of gallbladder cancer associated with the Mirizzi syndrome were incidentally found: a patient underwent right hepatectomy and another patient was unresectable. The overall morbidity rate was 16.6%. There was no postoperative mortality. An ERCP with stent insertion was required in three cases after surgery. Sixteen patients were asymptomatic at a mean distance of 24 months (range: 6-48) after surgery.

**Conclusions:**

Mirizzi syndrome requires being treated by an experienced biliary surgeon after a careful assessment of the local situation and anatomy. The preoperative placement of a stent via ERCP can simplify the surgical procedure.

## 1. Introduction

**Figure 1:**
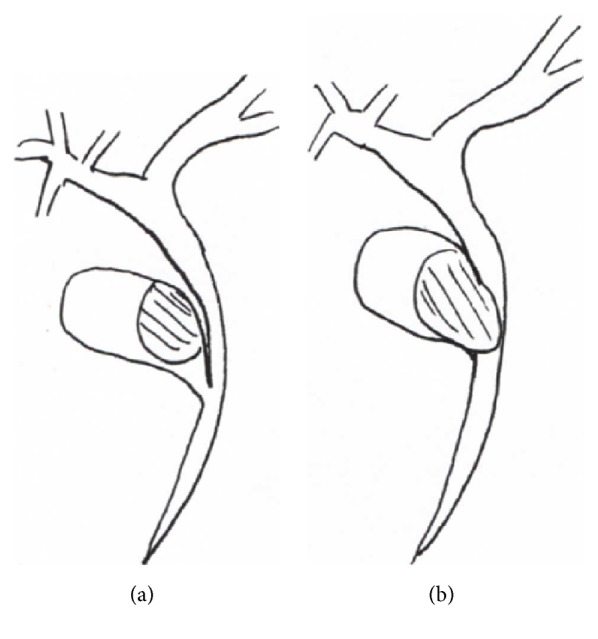
(a) Type I Mirizzi syndrome: a big stone impacted in the gallbladder infundibulum cause jaundice by extrinsic compression of the CBD; (b) type II Mirizzi syndrome: the stone is located in a single cavity formed by gallbladder and CBD.

Mirizzi syndrome was firstly reported by Pablo Luis Mirizzi (1893-1964), one of the major biliary surgeons in the past century. Mirizzi was born in Cordoba (Argentina) from Italian parents. He spent all his academic career in his hometown, where he left a deep mark [1, 2]. He is mainly known for conceiving and performing the first intraoperative cholangiography in 1931, a procedure that had a strong impact on biliary surgery of the XX century. Mirizzi first described his syndrome in 1948 [3], presenting the case of a patient with a big stone impacted in the gallbladder infundibulum, causing jaundice by extrinsic compression of the common bile duct (CBD) with a productive inflammation extending from the gallbladder to the CBD. This condition is an important complication of gallbladder stones and requires a differential diagnosis from gallbladder cancer involving the CBD. In some cases, the stone erodes from the gallbladder into the CBD determining a fistula; consequently, the stone is located in a single cavity formed by Hartmann's pouch and the CBD (Figure 1). In 1982 McSherry et al. named this condition “type II Mirizzi syndrome” [4], almost 20 years after Mirizzi's death. Type II represents the possible evolution of the properly mentioned Mirizzi syndrome (type I). In 1989 Csendes et al. [5] classified type II Mirizzi syndrome in three subgroups (II, III, and IV), considering the entity of the involvement of the CBD. In type II of Csendes classification, fistula involves 1/3 of the CBD circumference, in type III it involves the 2/3, and in the type IV the CBD is no more recognizable and represents a whole entity with the gallbladder. Recently, Béltran et al. [6], reporting several cases of Mirizzi syndrome associated with cholecysto-enteric fistula, classified these cases as type V, divided into two subgroups: (a) without gallstone ileus and (b) with gallstone ileus. Later, in response to a letter by Solis-Caxaj [7], Béltran [8, 9] simplified the classification of the syndrome identifying only three types:

(i) Type I, "classic" Mirizzi syndrome.

(ii) Type II cholecystocholedochal fistula with two subtypes: (a) diameter less than 50% of the CBD and (b) diameter more than 50% of the CBD.

(iii) Type III, with cholecysto-enteric fistula without (a) or with (b) gallstone ileus.

However, many authors continue to use the first classification of Csendes [5] which, by allowing a better distinction about the entity of the CBD wall involved in the fistula, could be useful to establish the right preoperative workup and surgical management.

This need for an accurate classification is related to the high frequency of the syndrome in South America, where it occurs in about 5% of gallstones, while in Western countries its incidence is about 1% [10].

Mirizzi syndrome is a condition difficult to diagnose and treat, representing a particular challenge for the biliary surgeon aware of the risk for the patient. The syndrome can mimic gallbladder cancer, but it also represents a precancerous condition, consequently causing considerable diagnostic difficulties. At the same time, because of the significant increase in the risk of intraoperative biliary injury during cholecystectomy, it is a very dangerous condition. At present, a standard treatment of Mirizzi syndrome is not yet well defined, due to the heterogeneous clinical presentation. Surgical treatment should be planned after a careful assessment of the local situation and anatomy. During the past five years we observed some consecutive cases of Mirizzi syndrome, which led us to review this field in an attempt to point out some particular aspects of diagnosis and treatment of this challenging condition.

## 2. Materials and Methods

Detailed clinical records of patients with diagnosis at discharge of Mirizzi syndrome, treated in our unit between January 1st, 2012, and December 31st, 2016, were reviewed. The following data were considered: sex and age of the patients; presenting symptoms and previous treatments; preoperative radiological investigations; preoperative diagnosis; surgical procedures performed; postoperative course (morbidity and mortality); findings at pathological examination. Follow-up data, obtained by direct clinical observation, laboratory findings (liver function tests), and need for further treatment, were also recorded.

## 3. Results

In the last 5 years, 18 consecutive cases of Mirizzi syndrome were treated in our unit and they represented 1.54% of 1.165 cholecystectomies performed in the same period. Eleven patients were male and 7 were female with a mean age of 63.4 years (range: 25-90 years). Presenting symptoms were obstructive jaundice in 14 patients, colic pain in 3 patients, and acute cholangitis in one patient. Preoperative imaging included ultrasonography (US) in all cases, Computerized Tomography (CT) in 12 patients, and Magnetic Resonance Imaging (MRI) in 10 patients. Fourteen patients underwent a preoperative ERCP; in all cases a stricture was found and, consequently, one or more stents were positioned in the CBD. Percutaneous cholangiography was performed in 2 patients and a percutaneous drainage was placed preoperatively. After diagnostic investigations, a diagnosis of Mirizzi syndrome was achieved in 11 patients, a diagnosis of gallbladder cancer was achieved in 6 patients, and a Klatskin tumor was diagnosed in one patient.

Seventeen patients underwent surgery. Eight patients with type I Mirizzi syndrome underwent a simple cholecystectomy, leaving in place the portion of the infundibulum adherent to the CBD. In three cases of Mirizzi type II, after cholecystectomy, a bile duct repair over T-tube was made. In other three cases of Mirizzi type II, an excision of the gallbladder and involved CBD with hepaticojejunostomy on Roux-en-Y loop was performed. The patient with type II Mirizzi presenting with acute colangitis was treated urgently with biliary drainage by ERCP and two months later underwent cholecystectomy and hepatico-jejunostomy. In patients with preoperative erroneous diagnosis of gallbladder cancer or Katskin's tumor, frozen sections and definitive histological examination clarified the diagnosis of Mirizzi syndrome. On the contrary, an unexpected gallbladder cancer associated with the Mirizzi syndrome was diagnosed intraoperatively in two patients: a 68-year-old man who received an explorative laparotomy for an unresectable cancer with peritoneal carcinomatosis and a 52-year-old female who underwent right hepatectomy with CBD excision for gallbladder cancer involving the CBD. Finally, a 90-year old man, who had initially received a diagnosis of gallbladder cancer, was re-evaluated with CT scan and MRI eighteen months later and was diagnosed as Mirizzi type-I syndrome. This patient did not undergo surgery and was treated by percutaneous drainage.

The overall morbidity rate was 16.6% (one case of post-ERCP pancreatitis and two cases of wound infection after surgery). There was no postoperative mortality. The 8 patients who underwent simple cholecystectomy enjoy good health with normal liver function tests. The three patients with CBD reconstruction over T-tube developed cholestasis after T-tube removal, due to the occurrence of a postoperative stricture: all cases were successfully treated with ERCP and multiple plastic stents insertions. The 4 patients who had undergone hepaticojejunostomy enjoy good health with normal liver function tests. The patient with unexpected finding of gallbladder cancer died three months after surgery. The patient who underwent right hepatectomy is alive six months after surgery and is being treated with chemotherapy. The patient with incorrect initial diagnosis of gallbladder cancer enjoys good health and replaces his percutaneous drainage at three month intervals. Clinical data are summarized in Table 1. All patients were monitored every six months after surgery (mean follow-up: 24 months, range: 6-48) by clinical evaluation, blood tests, and ultrasounds.

## 4. Discussion

The review of this clinical experience on Mirizzi syndrome allows some considerations regarding the diagnosis and treatment of the disease. In this series, patients with Mirizzi type I represented the majority of cases (11/18=61%). Preoperative diagnosis was correct in 11 cases out of 18 (61%), while the unexpected finding of cancer occurred in two cases (11%) a significant percentage, considering that unexpected cancer is usually found in less than 1% of cholecystectomies [11]. However, there is a complex relationship between Mirizzi syndrome and cancer: the high incidence of unexpected cancer is confirmed by the literature, as well as the erroneous preoperative diagnosis of cancer consequent to the particular clinical presentation [12, 13]. In our cases, a false diagnosis of cancer occurred in 39% of cases (7/18) explainable by the onset of jaundice in the absence of painful symptoms. The risk of bile duct injury during cholecystectomy for Mirizzi syndrome is increased according to the literature [14]. This fact is consequent to the anatomical difficulties during the dissection of the Calot's triangle.

**Figure 2:**
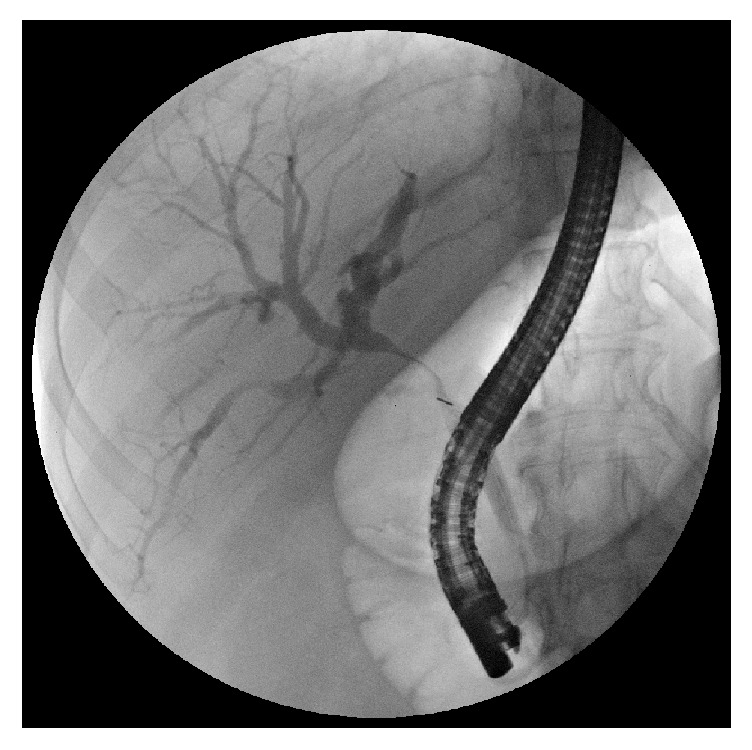
ERCP in a case of Mirizzi type I: note the smooth and regular stricture.

**Figure 3:**
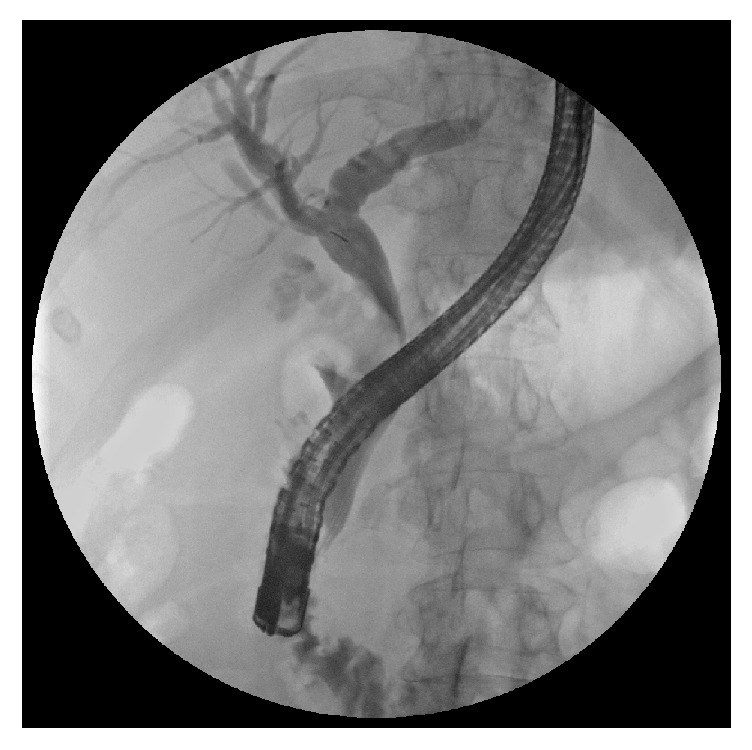
ERCP in a case of Mirizzi type II: filling defect of the CBD caused by a big stone.

**Figure 4:**
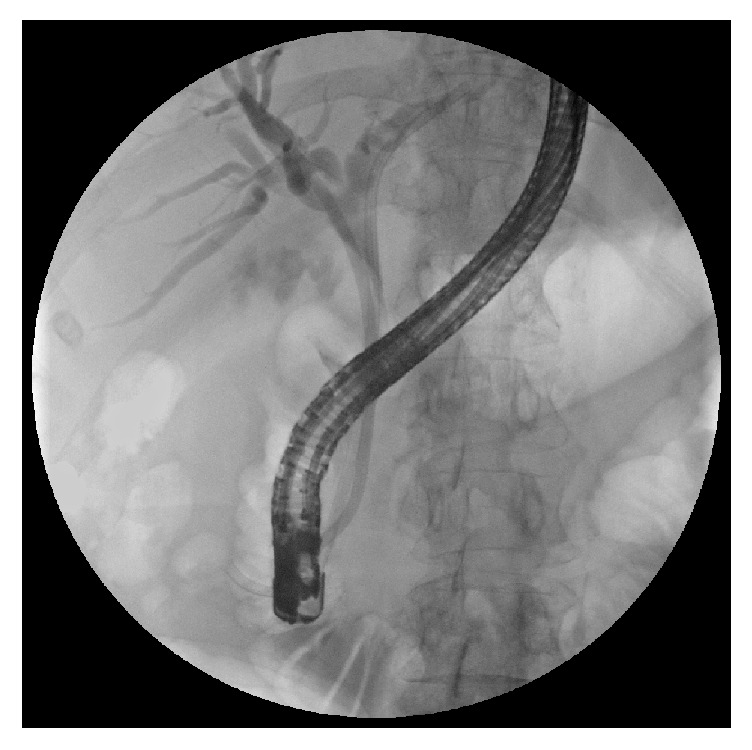
The same case after insertion of 2 stents.

**Figure 5:**
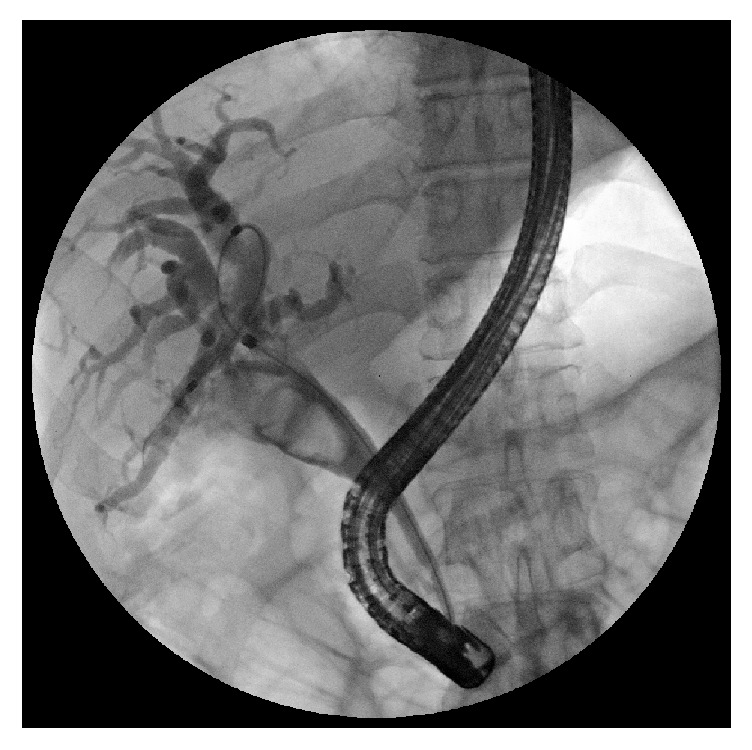
ERCP in a case of Mirizzi type II: there is a “common cavity” involving the gallbladder and the CBD.

Diagnostic difficulties are explained by the different clinical presentations of the disease. The classic presentation is obstructive jaundice without painful symptoms and with evidence, at ultrasonography, of a gallstone impacted in the gallbladder infundibulum and determining an external obstruction of the CBD with consequent dilation of the intrahepatic biliary tree. The diagnosis needs to be confirmed by CT or MRI and, finally, by surgery with histological examination. In these cases, in which it may be a Mirizzi syndrome type I or type IIa according to Béltran classification [10], it is suggested to perform an ERCP with the placement of one or more biliary plastic stents that simplify the surgical procedure by providing a protection to the CBD. Simple nasobiliary drainage does not eliminate this necessity. In addition, direct cholangiography obtained by ERCP provides a significant contribution to the correct diagnosis (Figures 2, 3, 4, and 5).

In type I Mirizzi syndrome, surgical treatment involves removal of the gallbladder leaving in place the portion of the infundibulum adherent to the CBD. In this way, the patency of the CBD is ensured by the presence of the stents. In type IIa Mirizzi syndrome the gallbladder is partially removed and part of the infundibular wall is used for the closure of the CBD. After surgery, stents can be eliminated spontaneously or subsequently removed. This modality of treatment is preferable to the CBD reconstruction over T-tube. In fact, after the removal of the T-tube, a stricture can appear requiring further endoscopic treatment, as in three cases of this series. The 4 cases of Mirizzi type IIb in our series received hepaticojejunostomy with an uneventful clinical outcome at follow-up. Regarding laparoscopic surgical approach, there are different opinions in the literature. Although laparotomy is considered a safer approach in the management of patients with Mirizzi syndrome, some authors [15, 16] report satisfactory results with laparoscopic technique, performing a subtotal cholecystectomy. However, the laparoscopic approach is recommended only to very experienced surgeons.

**Figure 6:**
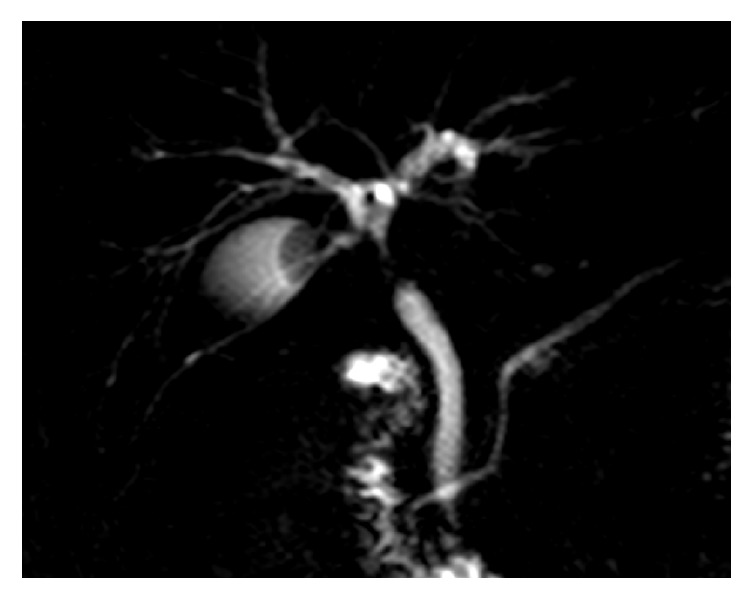
MRI cholangiography in a patient with Mirizzi type I mimicking a biliary cancer.

Another frequent clinical picture of the Mirizzi syndrome is that of a stricture mimicking biliary cancer. When the associated inflammatory process is predominant, it involves the CBD and mimics a neoplastic stricture at the MR-cholangiography (Figure 6). Patients usually undergo preoperative ERCP with placement of one or more stents. Endobiliary biopsies are obviously negative, taking into account the high frequency of false-negative findings. The diagnosis is clarified during surgical exploration by personal experience of the surgeon and analysis of the frozen-sections specimens. In these cases, the clinical onset and MRI findings can justify the preoperative diagnostic error. It should also be considered that up to 15% of suspicious biliary strictures are postoperatively found to be benign after surgery for Klatskin tumor [17]; the Mirizzi syndrome is one of the possible causes of this specific, sometimes unavoidable, diagnostic error.

A clinical onset with acute cholangitis is typical of type II Mirizzi syndrome: in these cases, diagnosis is facilitated by ERCP that allows to reduce the pressure in the CBD by positioning of nasobiliary drains or stents. However, surgery still represents the definitive solution: the choice between cholecystectomy with fistula closure and hepaticojejunostomy should be evaluated by the surgeon taking into account the extent of the CBD involvement, according to the Béltran categories [10].

In some cases, the Mirizzi syndrome can represent an unexpected intraoperative finding and the main risk is accidental bile duct injury. This happens when the surgeon wants to complete the cholecystectomy “at any cost” without previous identification of the CBD. On the contrary, the main goal must be to avoid any injury to the bile ducts and, therefore, the placement of a cholecystostomy may be an appropriate and justified measure as a bridge solution before referring the patient to a hepatobiliary center. Alternatively, it may be carried out a subtotal cholecystectomy, leaving in place a portion of the infundibulum adherent to the CBD, after removing the stones. Testini and coworkers suggested a decision algorithm for emergency in nonspecialized centers [18].

## 5. Conclusions

Mirizzi syndrome continues to be a disease of difficult diagnosis and treatment. The general surgeon without long experience in hepatobiliary surgery should refer the patient to a specialized hepatobiliary surgical center. It is difficult to standardize the treatment of the disease since clinical presentation and anatomical situations are very variable (types I or II, suspected carcinoma). It is important, however, for surgeons to know the disease and the possible intraoperative challenging situations. In patients with Mirizzi syndrome type I, the best management seems to be the preliminary placement of one or more stents by ERCP, followed by cholecystectomy leaving in place a small portion of the gallbladder adherent to the CBD. It is advisable to leave the stent in the postoperative period and remove it after nearly 2 months. In the occurrence of a postoperative stricture, endoscopic treatment with positioning of multiple plastic stents is recommended until resolution. In Mirizzi syndrome type II subtype a (according to Béltran classification), treatment may be similar to type I; however, a close attention to the diameter of the residual CBD is required during the reconstructive phase. In type IIb, the definitive solution appears to be hepaticojejunostomy. As for the type of surgical approach (laparoscopy or laparotomy), laparoscopy is reserved to a very experienced surgeon. However, in our opinion, laparotomy allows better evaluation of biliary anatomy, avoiding any risk of bile duct injury and with the advantage of a more accurate surgical procedure.

## Figures and Tables

**Table 1:** Clinical data summarized.

Pt	Sex	Age	Symptom	Preop. Imaging	Preop. diagnosis	Treatment	Final diagnosis
1	M	64	Cholangitis	US, ERCP	Mirizzi	Hepatico-jejunostomy	Mirizzi type II

2	M	79	Jaundice	US,CT,ERCP	Gallbladder cancer	Cholecystectomy	M. type I

3	M	82	Pain	US, MRI,ERCP	Mirizzi	Cholecystectomy	M. type I

4	M	90	Jaundice	US,CT,MRI,PTC	Gallbladder cancer	Nonoperative, PTC	M. type I

5	M	78	Jaundice	US,CT	Klatskin's tumor	Cholecystectomy	M. type I

6	M	61	Jaundice	US,CT,MRI,ERCP	Mirizzi	Cholecystect. + T-tube	Mirizzi type II

7	F	65	Jaundice	US,MRI,ERCP	Mirizzi	Cholecystectomy	M. type I

8	M	72	Jaundice	US,ERCP	Gallbladder cancer	Hepatico-jejunostomy	Mirizzi type II

9	M	67	Pain	US,CT,MRI,ERCP	Mirizzi	Cholecystectomy	M. type I

10	F	65	Jaundice	US,CT,ERCP	Mirizzi	Cholecystectomy	M. type I

11	F	38	Jaundice	US,CT,MRI,PTC	Gallbladder cancer	Hepatico-jejunostomy	Mirizzi type II

12	M	68	Jaundice	US,CT,MRI,ERCP	Mirizzi	Explor.Laparotomy	M. type I + GBC

13	F	56	Jaundice	US,CT,ERCP	Mirizzi	Cholecystect. + T-tube	Mirizzi type II

14	F	56	Pain	US,CT,ERCP	Mirizzi	Cholecystect. + T-tube	Mirizzi type II

15	F	56	Jaundice	US,CT,MRI	Gallbladder cancer	Hepatico-jejunostomy	Mirizzi type II

16	M	68	Jaundice	US,CT,ERCP	Gallbladder cancer	Cholecystectomy	M. type I

17	M	25	Jaundice	US,MRI,ERCP	Mirizzi	Cholecystectomy	M. type I

18	F	52	Jaundice	US,MRI,ERCP	Mirizzi	Right hepatectomy	M. type I + GBC

*Note.* US=ultrasonography; CT=Computerized Tomography; MRI= magnetic resonance imaging; PTC= percutaneous transhepatic cholangiography; ERCP= endoscopic retrograde cholangiopancreatography; GBC= gallbladder cancer.

## Data Availability

All clinical data are listed in the table and are available for consultation.
